# Role of Glutamatergic Excitotoxicity in Neuromyelitis Optica Spectrum Disorders

**DOI:** 10.3389/fncel.2019.00142

**Published:** 2019-04-12

**Authors:** Ana Paula Bornes da Silva, Débora Guerini Souza, Diogo Onofre Souza, Denise Cantarelli Machado, Douglas Kazutoshi Sato

**Affiliations:** ^1^Molecular and Cellular Biology Laboratory, Brain Institute, Pontifical Catholic University of Rio Grande do Sul (PUCRS), Porto Alegre, Brazil; ^2^Medical School, Institute of Geriatrics and Gerontology, Graduate Program in Biomedical Gerontology, Pontifical Catholic University of Rio Grande do Sul (PUCRS), Porto Alegre, Brazil; ^3^Graduate Program in Biological Sciences: Biochemistry, Federal University of Rio Grande do Sul (UFRGS), Porto Alegre, Brazil

**Keywords:** neuromyelitis optica spectrum disorders, aquaporin-4, antibody, astrocytes, glutamate, excitotoxicity

## Abstract

Neuromyelitis optica spectrum disorder (NMOSD) is an inflammatory disorder mediated by immune-humoral responses directed against central nervous system (CNS) antigens. Most patients are positive for specific immunoglobulin G (IgG) auto-antibodies for aquaporin-4 (AQP4), a water channel present in astrocytes. Antigen-antibody binding promotes complement system cascade activation, immune system cell infiltration, IgG deposition, loss of AQP4 and excitatory amino acid transporter 2 (EAAT2) expression on the astrocytic plasma membrane, triggering necrotic destruction of spinal cord tissue and optic nerves. Astrocytes are very important cells in the CNS and, in addition to supporting other nerve cells, they also regulate cerebral homeostasis and control glutamatergic synapses by modulating neurotransmission in the cleft through the high-affinity glutamate transporters present in their cell membrane. Specific IgG binding to AQP4 in astrocytes blocks protein functions and reduces EAAT2 activity. Once compromised, EAAT2 cannot take up free glutamate from the extracellular space, triggering excitotoxicity in the cells, which is characterized by overactivation of glutamate receptors in postsynaptic neurons. Therefore, the longitudinally extensive myelitis and optic neuritis lesions observed in patients with NMOSD may be the result of primary astrocytic damage triggered by IgG binding to AQP4, which can activate the immune-system cascade and, in addition, downregulate EAAT2. All these processes may explain the destructive lesions in NMOSD secondary to neuroinflammation and glutamatergic excitotoxicity. New or repurposed existing drugs capable of controlling glutamatergic excitotoxicity may provide new therapeutic options to reduce tissue damage and permanent disability after NMOSD attacks.

## Introduction

The central nervous system (CNS) is the target of several pathologies, including CNS autoimmune diseases, a diversified class of disorders that target neuronal and glial antigens. These disorders may be syndromes associated with auto-antibodies that attack intracellular neural antigens or surface antigens. Among the surface antigens, the membrane proteins present on the neuronal or glial surface might be important targets. In the CNS, the antigen-antibody interaction compromises the homeostasis of the system and may cause damage to neural cells (Zettl et al., 2012; Höftberger, 2015).

Neuromyelitis optica spectrum disorder (NMOSD) is an inflammatory disorder mediated by immune-humoral responses directed against CNS antigens (Sato et al., 2013). Most patients are positive for serum antibodies that target the water channel aquaporin-4 [AQP4–immunoglobulin G (IgG)], a water channel expressed in the end-feet of astrocytes (Wingerchuk et al., 2006, 2015). This inflammatory context is signaled by activated T cells, which cross the cerebral vascular endothelium and impair blood-brain barrier (BBB), promoting the migration of other inflammatory cells, such as macrophages and granulocytes, into the brain and spinal cord tissue (Kurosawa et al., 2015). Moreover, the binding between IgG and AQP4 triggers exacerbation of astrocytic lesions characterized by massive loss of AQP4 and consequent tissue damage that can lead to secondary demyelination due to oligodendrocyte destruction (Kurosawa et al., 2015; Li and Yan, 2015; Zeka et al., 2015; Zekeridou and Lennon, 2015). The lesions are predominantly localized on the optic nerves and spinal cord, compromising the visual and motor capacity of NMOSD patients (Zeka et al., 2015; Zekeridou and Lennon, 2015).

Astrocytes are the main cells regulating glutamatergic homeostasis and, once injured, their ability to perform physiological functions becomes impaired (Zeng et al., 2007; Haruki et al., 2013; Yang et al., 2016). Some studies suggest that the death of oligodendrocytes and secondary demyelination lesions after astrocyte injury may be related to high extracellular concentrations of the neurotransmitter glutamate in the CNS tissue (reviewed by Yang et al., 2016). Neurons are also highly sensitive to high glutamate concentrations, and glutamate excitotoxicity may promote neuronal death, increasing the risk of disability (Marignier et al., 2010; Haruki et al., 2013). The aim of this review is to discuss the relationship between astrocyte damage and glutamatergic excitotoxicity in AQP4-IgG-positive NMOSD.

## The Role of Astrocytes in the Brain

**Figure 1:**
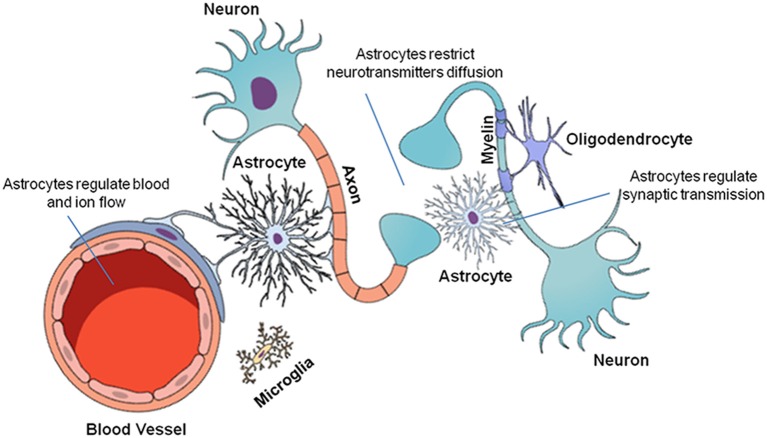
Cellular organization of the central nervous system (CNS). The CNS is composed of neuronal and glial cells (microglia, oligodendrocytes, and astrocytes). Each cell performs a specific function in the CNS. Neurons transmit chemical and electrical signals to other nerve cells. Microglia are immune system cells responsible for the defense of the CNS. Oligodendrocytes form the myelin sheaths of the axons, facilitating saltatory nervous signal conduction. Finally, astrocytes are multifunctional cells that control ion and neurotransmitter diffusion in the nervous parenchyma, in addition to actively participating in the synapses and providing metabolic support to the other cells.

The CNS is composed of neurons and glial cells (Figure 1). Glial cells perform critical functions in the CNS, such as modulating and eliminating synapses, supporting neurons with energetic sources and playing an immune role (Allen and Barres, 2009). Glial cells are as numerous as neurons (Allen and Barres, 2009; Gutiérrez Aguilar et al., 2017). This group of cells includes microglia, oligodendrocytes and astrocytes. Their unique biochemical and molecular features allow them to play pivotal roles in CNS physiology (Domingues et al., 2016).

Astrocytes are particularly important among the glial cells since they participate in information processing in the brain from the early stages of development and throughout adult life (Allen and Barres, 2009). They perform various functions in the brain, such as controlling the balance of extracellular ions and water through specialized transmembrane proteins, including AQP4 and ion channels (Papadopoulos and Verkman, 2013; Verkman et al., 2013). Astrocytes can act in CNS repair, maintain BBB homeostasis and regulate the extracellular ionic content to allow an action potential to occur (Maragakis and Rothstein, 2006; Iacovetta et al., 2012; Domingues et al., 2016; Hubbard et al., 2018). Additionally, they secrete growth factors that stimulate surrounding cells, such as molecules that directly influence synapse formation, which is essential in synaptic modulation and synaptic plasticity (Crawford et al., 2012; Fang et al., 2012).

Astrocytes are anatomically associated with neuronal cell bodies and synapses. Therefore, they can regulate the chemical content of the extracellular space and restrict the diffusion of neurotransmitters released into the synaptic cleft (Tritsch and Bergles, 2007; Allen and Barres, 2009; Gutiérrez Aguilar et al., 2017). The classical example is the modulation of the glutamate extracellular concentration through excitatory amino acid transporters (EAATs)/high-affinity glutamate transporters expressed in astrocytic cell membranes (Danbolt, 2001; Danbolt et al., 2016). These cells are responsible for removing glutamate from the extracellular space to promote cerebral homeostasis and prevent excitotoxicity (Benediktsson et al., 2012; Vasile et al., 2017). Therefore, they can regulate extracellular concentrations of substances that can potentially interfere in normal brain functions (Domingues et al., 2016; Hubbard et al., 2018). Recent findings suggest that glial cells are a target of several pathologies. The role of glial cells in health and disease is slowly being elucidated, and the interactions of glia with other CNS cells seem to play fundamental roles in brain performance during normal development and disease states (Domingues et al., 2016).

## Cerebral Glutamate

Glutamate is the most abundant CNS excitatory molecule and the main neurotransmitter in mammalian brain (Rose et al., 2018). Glutamate excites nerve cells, especially neurons, thus playing a key role in cell signaling (Zhou and Danbolt, 2014). Glutamate is known to be active in several brain processes, such as cognition, memory and learning. In addition, it plays an important role in the induction and elimination of synapses and contributes to cell migration, differentiation and death (Danbolt, 2001). Glutamate also participates in the synthesis of proteins, peptides, and purines (Hackett and Ueda, 2015) and plays a fundamental role in amino acid metabolism (Skytt et al., 2012). Glutamate signaling has been extensively studied, especially due to its vital role in brain normal function and due to its association with pathologies that affect the CNS (Fang et al., 2012; Lewerenz and Maher, 2015).

Glutamate is stored in vesicles at the presynaptic terminal through the action of vesicular glutamate transporters (vGLUTs) and released in the synaptic cleft after presynaptic membrane depolarization. When glutamate is released, it binds to ionotropic glutamate receptors (iGluRs), such as N-methyl-D-aspartate (NMDA) receptors, α-amino-3-hydroxy-5-methyl-4-isoxazole propionic acid (AMPA) receptors, kainate receptors and metabotropic receptors on the postsynaptic membrane, as well as metabotropic receptors on the presynaptic membrane (Reiner and Levitz, 2018). This depolarization excites the postsynaptic neuron, generating an action potential in the axon to carry the nerve signal (Zhou and Danbolt, 2014; Lewerenz and Maher, 2015; Hayashi, 2018).

The interaction of glutamate with its specific receptors produces postsynaptic excitatory potentials in a precise and controlled manner (Danbolt, 2001; Reiner and Levitz, 2018). The glutamate extracellular concentrations must be well controlled because high glutamate concentrations excessively activate its receptors, generating oxidative stress that may lead to cell death, a process known as excitotoxicity, which impairs both neurons and glial cells (Zhou and Danbolt, 2013, 2014; Stojanovic et al., 2014), causing synaptic transmission dysfunction and interfering in synaptic plasticity (Pál, 2018). For such modulation, glutamate concentrations are controlled by astrocytes through cellular reuptake since these cells present specific proteins in their cell membranes that take up free glutamate as we discuss below (Danbolt, 2001; Larsson et al., 2004; Benarroch, 2010). Glutamate present in the extracellular space cannot be metabolized by any other mechanism (Zhou and Danbolt, 2013). Therefore, astrocytes are essential in the modulation of the glutamatergic system, and any impairment in their highly coordinated action may contribute to the onset of pathologies (Miladinovic et al., 2015).

## Astrocytes and Glutamate Transporters

Astrocytes express EAATs in their cell membranes, which are also known as glutamate transporters (Lewerenz and Maher, 2015; Al Awabdh et al., 2016). These transporters can retain excess glutamate from the extracellular space inside the cell, thus potentially avoiding excitotoxicity (Gasparini and Griffiths, 2013). Therefore, they transport glutamate in cells against their intra- and extracellular concentration gradients, contributing to the low extracellular glutamate concentration (Lewerenz and Maher, 2015; Underhill et al., 2015; Gutiérrez Aguilar et al., 2017). Glutamate transporters also contribute to physiological synaptic plasticity and function (Rose et al., 2018) and can function as chloride channels (Wadiche et al., 1995a,b; Wadiche and Kavanaugh, 1998) and water transporters (MacAulay et al., 2001, 2004). Five glutamate transporters belonging to the solute-1 carrier family have been identified (Benarroch, 2010; Jiang and Amara, 2011). These transporters are expressed in various tissues, but their main contribution is control of excitatory neurotransmission in brain tissue (Grewer et al., 2014). EAAT1 and EAAT2 are highly expressed in astrocytes, EAAT3 is expressed in neurons (Lewerenz and Maher, 2015; Hayashi, 2018; Schousboe, 2018), EAAT4 is present in the dendritic spines of Purkinje cerebellar cells, and EAAT5 is the retinal glutamate transporter (Gutiérrez Aguilar et al., 2017; Pál, 2018).

Astrocytes are well documented to be the cells responsible for detoxification of metabolic waste and extracellular ions and molecules, such as glutamate (Rose et al., 2018). When glutamate enters in the astrocytic compartment, it can be degraded, recycled or transported out of the brain through the blood or gliolymphatic system (Rose et al., 2018). In the adult brain, approximately 80%–90% of extracellular glutamate is captured by EAAT2 (Danbolt et al., 2016). In addition to glutamate, these transporters cotransport three Na^+^ molecules and one proton (H^+^) with each glutamate molecule. Additionally, this system is coupled to the reverse transport of one K^+^ (Zerangue and Kavanaugh, 1996). Thus, an electrochemical gradient is created on the plasma membrane, allowing transporters to maintain low concentrations of extracellular glutamate (Bergles et al., 2002; Larsson et al., 2004; Lewerenz and Maher, 2015).

**Figure 2:**
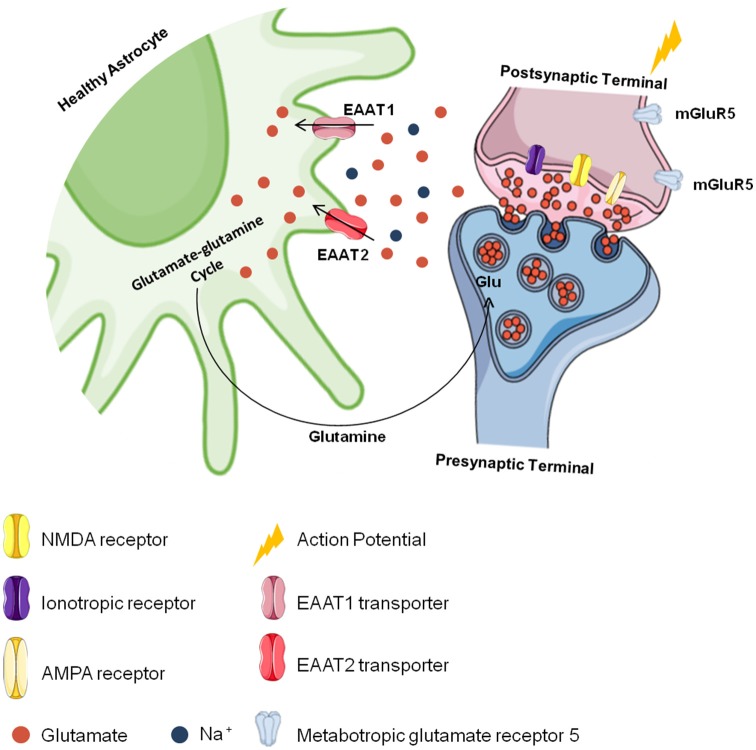
Cerebral glutamate flow. Glutamate is stored presynaptically in vesicles by vesicular glutamate transporters (vGLUTs). It is released after presynaptic membrane depolarization, binds to ionotropic glutamate receptors (iGluRs). like N-methyl-D-aspartate (NMDA) and α-amino-3-hydroxy-5-methyl-4-isoxazole propionic acid (AMPA) receptors in the postsynaptic membrane and generates an action potential. The excess glutamate released into the synaptic cleft is regulated by astrocytes through the excitatory amino acid transporter 1 (EAAT1) and EAAT2 transporters against a concentration gradient. In astrocytes, glutamate is recycled and converted to glutamine, which is transported to neurons and converted into glutamate again to be used in a new synapse.

Astrocytes also convert glutamate into glutamine through the glutamine synthetase reaction (Norenberg and Martinez-Hernandez, 1979; Jayakumar and Norenberg, 2016), and glutamine is later transported to neurons and converted back to glutamate to be used again in neurotransmission in a process known as the “glutamate-glutamine cycle” (Grewer et al., 2014; Lewerenz and Maher, 2015; Hayashi, 2018; Pál, 2018; Schousboe, 2018; Figure 2). Since they regulate glutamatergic signaling, glutamate transporters are essential to brain metabolism (Fang et al., 2012; Grewer et al., 2014; Lewerenz and Maher, 2015). As EAAT2 plays an important role in the physiological functioning of the brain, it is believed to also play a role in the development of chronic and acute CNS disorders (Soni et al., 2014; Grewer et al., 2014). Studies with simultaneous EAAT1- and EAAT2-knockout animals show that they are nonviable because of brain abnormalities and cortical disorganization (Ciappelloni et al., 2017; Rose et al., 2018). Animals deficient only in EAAT2 die after birth due to seizures (Tanaka et al., 1997; Al Awabdh et al., 2016; Danbolt et al., 2016). In adults, impairment in EAAT2 function has been observed to compromise cerebral glutamatergic homeostasis (Hayashi, 2018; Rose et al., 2018). Taken together, the loss of glutamate transporters in astrocytes may contribute to CNS dysfunction and increase neuronal damage in focal inflammatory lesions.

Considering the aforementioned roles of astrocytes and glutamate transporters, understanding astrocyte injury in NMOSD and these entities’ contributions to the glutamatergic excitotoxicity observed in this condition is vital since astrocytic impairment can cause serious consequences in brain homeostasis and reduce synaptic function, contributing to the NMOSD physiopathology (Hinson et al., 2008). For these reasons, astrocytic damage in NMOSD is believed to trigger an imbalance in glutamatergic homeostasis, which may contribute to the formation of longitudinally extensive myelitis and optic neuritis lesions (Haruki et al., 2013; Yang et al., 2016).

## Glutamate in NMOSD

Most patients with NMOSD produce IgG auto-antibodies that are highly specific for AQP4, but the cellular and molecular mechanisms of this interaction are still not clear. Damage occurring in other cells of the CNS, such as neurons and oligodendrocytes, is assumed to be due to a primary astrocytic lesion (Hinson et al., 2008). Astrocytes have been studied for a few decades in both *in vivo* and *in vitro* models of various diseases. Cultivation of astrocytes as primary cultures and lineages represents a powerful tool to explore specific information provided by these cells and to reveal mechanisms related to their function (Hertz et al., 1998). The brain is a complex system in which several cell types interact. While some regions are enriched in a specific cell subtype, an isolated cell type does not occur naturally. Regardless, the use of *in vitro* models of specific cell types has been very helpful in the progress of the neuroscience field (Lange et al., 2012). *In vitro* models of NMOSD have shown that auto-antibodies against AQP4 protein trigger a phenomenon known as antigenic modulation because when such auto-antibodies bind to their targets, a specific IgG alters the functions of AQP4 through its degradation or internalization by astrocytes and interferes in sodium-potassium-dependent glutamate uptake (Hinson et al., 2010). For this reason, *in vitro* NMOSD models are important for identifying cellular interactions that cause nerve tissue damage in this disease.

Such models basically consist of rodent/human astrocyte cultures or immortalized lineages that are exposed to AQP4+ serum samples or only purified IgG, with or without the addition of a human complement, which is used to evaluate the possible deleterious effects of related conditions (Haruki et al., 2013). *In vitro* assays have shown that auto-antibodies present in serum derived from NMOSD patients are cytotoxic and harmful to astrocytes, modifying their morphology and function (Li and Yan, 2015). These conditions induce the internalization of AQP4 protein and consequently other membrane proteins, such as EAAT2, since AQP4 protein forms complexes with other membrane proteins, including glutamate transporters (Zeng et al., 2007; Haruki et al., 2013; Yang et al., 2016).

Haruki et al. (2013) showed that when astrocyte cultures are exposed to AQP4-IgG+ human serum or purified IgG, these cells undergo morphological alterations, with compression of their cell bodies and reduction of cell processes, both in the presence and absence of a human complement. This finding has been confirmed by cell viability assays showing that cells treated with NMOSD patient samples have reduced survival rates compared to cells treated with serum from healthy controls. Astrocyte cultures exposed to AQP4-IgG+ NMOSD patient samples showed reduced EAAT2 transporter expression on cell membranes, suggesting that under these conditions, the AQP4-IgG complex exerts an indirect negative effect on this transporter, impairing its physiological role (Haruki et al., 2013).

**Figure 3:**
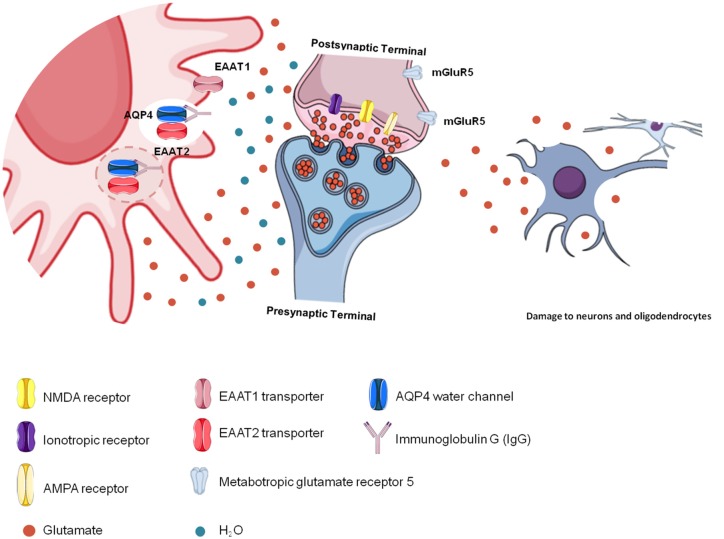
Glutamatergic toxicity in neuromyelitis optica spectrum disorder (NMOSD). Aquaporin-4 (AQP4)-immunoglobulin G (IgG) auto-antibodies binding to AQP4 may promote the internalization of the water channel, thus affecting cellular hydric homeostasis. Antigen-antibody binding promotes immune activation but also affects glutamatergic homeostasis because of reduced glutamate transporter (EAAT2) activity due to its internalization together with AQP4. In the absence of EAAT2, glutamate remains free in the extracellular space, promoting glutamatergic toxicity, which affects other cells, such as neurons and oligodendrocytes.

NMOSD is characterized by astrocyte death due to binding of the AQP4-IgG complex to AQP4 in astrocyte membranes (Wingerchuk et al., 2015). For this reason, astrocytes cannot capture free glutamate from the extracellular space, causing excitotoxicity and damaging other nerve cells and their cellular structures, such as the myelin sheath, as observed in longitudinally extensive myelitis lesions and optic neuritis (Hinson et al., 2008). Therefore, impairment in glutamatergic homeostasis induces excitotoxicity in neurons and oligodendrocytes, promoting the destruction of myelin (Stojanovic et al., 2014; Figure 3).

Excitotoxicity is evident in important CNS disorders and may also be present in NMOSD (Lewerenz and Maher, 2015). *In vitro* studies show that after IgG binding to AQP4, a decrease in the EAAT2 content occurs in the cell membrane, with a consequent decrease in the ability of cells to take up glutamate (Hinson et al., 2017). Since astrocytes are damaged in NMOSD lesions and EAAT2 is not functional, these cells are unable to take up glutamate. Thus, the lesions of longitudinally extensive myelitis and optic neuritis observed in patients with NMOSD may contribute to excess extracellular glutamate since astrocytes are damaged and lose their functionality (Hinson et al., 2008, 2017). Oligodendrocytes and neurons are highly sensitive to extracellular glutamate accumulation, and lesions in NMOSD are characterized by loss of the myelin sheaths that line the neurons, which are produced by oligodendrocytes. Excess glutamate in the extracellular space may contribute to neurotoxic events that lead to oligodendrocyte dysfunction and consequent demyelination (McDonald et al., 1998; Hinson et al., 2008, 2010).

## AQP4-IgG Downregulates EAAT2

Aquaporins are membrane proteins responsible for cellular water balance (Papadopoulos and Verkman, 2013; Verkman et al., 2013; Nakada, 2015). Thirteen proteins have been identified in various mammalian species and are distributed throughout the organism, including the brain (Iacovetta et al., 2012). In the brain, the most expressed aquaporin is AQP4, which can be found in the cerebral cortex, corpus callosum, retina, optic nerves, cerebellum, hypothalamus magnocellular nucleus and brainstem (Papadopoulos and Verkman, 2013; Ikeshima-kataoka, 2016). AQP4 is vital for the brain since it also regulates potassium uptake and release by astrocytes and facilitates cell migration, glial scar formation and cellular communication (Papadopoulos and Verkman, 2013; Verkman et al., 2013; Hubbard et al., 2018). Studies using AQP4-knockout animals have shown that this protein facilitates the movement of water into and out of the CNS. In the absence of AQP4, animals show cytotoxic brain edema due to an osmotic imbalance (Verkman et al., 2013, 2017).

AQP4 forms complexes with other membrane proteins, such as glutamate transporters, especially EAAT2, and can regulate this transporter positively when it is functional or negatively when it is damaged (Chaudhry et al., 1995; MacAulay et al., 2002; Queen et al., 2007; Zeng et al., 2007; Hinson et al., 2008; Xing et al., 2017). Thus, the interaction between IgG and AQP4, which is known as the “antigen-antibody complex” (AQP4-IgG), compromises EAAT2 function, resulting in excitotoxicity and impaired CNS cell function (Zeng et al., 2007; Fang et al., 2012; Haruki et al., 2013; Lewerenz and Maher, 2015; Yang et al., 2016). The AQP4-IgG complex induces downregulation of EAAT2, resulting in high concentrations of glutamate in the brain (Mattson, 2003; Park et al., 2004; Hinson et al., 2010). This process increases extracellular glutamate concentrations, aggravating typical NMOSD lesions associated with the complement system, and provides a cytotoxic environment for neurons and oligodendrocytes (Hinson et al., 2010).

Accordingly, Haruki et al. (2013) showed that EAAT2 has diminished expression in human adult astrocyte lineages exposed to specific IgG, suggesting that the AQP4-IgG complex downregulates this transporter in NMOSD. Astrocyte cultures derived from AQP4-knockout animals show low EAAT2 expression as well as a low astrocytic ability to take up glutamate (Zeng et al., 2007). Marignier et al. (2010) observed that purified IgG from patients with NMOSD reduces the number of AQP4+ cells, and the remaining cells exhibit low glutamate uptake. Therefore, in this model, we can conclude that the AQP4-IgG complex results in the loss of EAAT2 and consequently impacts glutamatergic homeostasis. Subsequently, damage to neurons and oligodendrocytes is observed, causing the classic lesions that characterize NMOSD (Marignier et al., 2010; Haruki et al., 2013).

In a glutamate uptake assay, Hinson et al. (2008) observed that cells take up little glutamate (less than 50%) after AQP4+ serum exposure, and EAAT2 expression is as low as AQP4 expression, undetectable by immunofluorescence, after serum exposure. In addition, immunohistochemical assays of human CNS tissue (the cortex and spinal cord) showed that EAAT2 is normally colocalized with AQP4 protein in astrocytes of the gray matter (Chaudhry et al., 1995). The same type of colocalization has also been observed in rodents (Queen et al., 2007), corroborating the hypothesis that AQP4 can form protein complexes and exerts regulatory action on GLT-1 (EAAT2 analogous in rodents) due to the action of the specific IgG (Hinson et al., 2008, 2017).

Under normal physiological conditions, EAAT2 is known to be enriched in the spinal cord (Nakamura et al., 2008). In NMOSD, this tissue is extensively damaged, and the resultant lesions can be explained when considering the theory that AQP4-IgG downregulates EAAT2 (Hinson et al., 2008); therefore, the loss of this transporter may contribute to the destructive lesions of the spinal cord observed in patients with NMOSD. Since the lesions are necrotic, NMOSD lesions may emerge not only from complement activation but also due to the negative control exerted by AQP4-IgG on EAAT2, preventing glutamatergic homeostasis (Wingerchuk et al., 2007; Hinson et al., 2008, 2010; Newcombe et al., 2008). Therefore, impairment of AQP4 functions through AQP4-IgG binding decreases EAAT2’s regulation of extracellular glutamate, resulting in glutamatergic excitotoxicity that promotes the death of other nerve cells and contributes to the formation of lesions characteristic of NMOSD (Yang et al., 2016).

## Therapeutic Interventions

The drug therapies developed to date to treat autoimmune diseases such as NMOSD aim to reduce the inflammatory process through immunosuppression. However, new oral therapies using small molecules that are directly permeable to the BBB may be promising for neuroprotection (Luchtman et al., 2016), so both immunosuppressive and neuroprotective drugs may be combined to treat patients. Therefore, investigating drugs that modulate astrocyte function and glutamate uptake and have a protective effect on other cells may contribute to the treatment of several CNS neuroinflammatory pathologies, such as NMOSD.

Antigen-antibody binding in NMOSD promotes the loss of important astrocyte functions associated with AQP4 and EAAT2, which leaves glutamate at high concentrations in the extracellular space. Considering that excess glutamate contributes to the formation of spinal cord and optic nerve lesions, a therapy that regulates glutamatergic excitotoxicity could be useful to prevent such lesions in NMOSD. Studies with rodents show that ceftriaxone, a beta-lactam antibiotic that increases EAAT2 expression, thus facilitating the removal of free glutamate and preventing glutamatergic excitotoxicity (Hsu et al., 2015), positively regulates EAAT2 acutely and chronically (Rothstein et al., 2005; Szu and Binder, 2016; Zimmer et al., 2017), thus providing neuroprotection under excitotoxic stress conditions. In *in vitro* models derived from spinal cord cultures, ceftriaxone reduces neuronal loss by increasing EAAT2 expression (Bajrektarevic and Nistri, 2017).

Bajrektarevic and Nistri (2017) observed that excitotoxic stress induction with 100 μM kainate promotes EAAT2 immunoreactivity in astrocyte cultures pretreated with ceftriaxone for 3 days, suggesting that ceftriaxone confers neuroprotection against an excitotoxic stimulus/challenge when administered prior to treatment with a glutamate uptake inhibitor. In an experimental model of Parkinson’s disease, ceftriaxone has been shown to increase EAAT2 expression in the hippocampus, regardless of whether the treatment started before or after the injury. This drug crosses the BBB and can penetrate into the CNS at therapeutic levels. In the context of NMOSD, ceftriaxone may be studied to determine whether it can reduce acute longitudinally extensive myelitis and optic neuritis lesions based on reduced cell damage secondary to excess glutamate (Hsu et al., 2015).

The deleterious effect of glutamate has already been elucidated in several neurodegenerative diseases such as Alzheimer’s. Memantine is a neuroprotective drug recommended for the treatment of Alzheimer’s disease, and as a noncompetitive NMDA receptor antagonist, this drug reduces receptor affinity for glutamate (Matsunaga et al., 2018). Memantine may confer neuroprotection through more potent inhibition of extrasynaptic NMDA receptors (Zhao et al., 2006; Léveillé et al., 2008; Okamoto et al., 2009; Milnerwood et al., 2010; Xia et al., 2010) without significantly affecting physiologic glutamatergic transmission. In addition, this drug has few undesirable drug interactions or adverse effects and is a well-tolerated medication for neurological disorders (Seyedsaadat and Kallmes, 2019). Several studies have investigated the effects of memantine on neurons, and the drug is known to increase the release of glial cell neurotrophic factors, contributing to neuronal survival (Wu et al., 2009). Further, the anti-inflammatory action of memantine reduces pro-inflammatory factors (reactive oxygen species and tumor necrosis factor-α), inhibiting the activation of microglia, and may provide neuroprotection (Maciulaitiene et al., 2017).

In addition to Alzheimer’s disease, in retinal crush models (a classic glaucoma model), astrocytes treated with intravitreal memantine have been found to exhibit improved survival (Maciulaitiene et al., 2017). Memantine preserves retinal astrocytes by exerting a glioprotective effect. In glaucoma models, an altered ion concentration in cells causes cellular damage due to high sodium influx and elevated intracellular calcium, causing glutamate release and cell death. Under memantine treatment, this process is attenuated, preventing excitotoxicity-induced damage to nerve cells. Memantine, as a noncompetitive NMDA receptor blocker, can reduce the optic neuritis observed in patients with NMOSD as well as retinal damage in some cases (Maciulaitiene et al., 2017; Bradl et al., 2018). In addition to its protective effect, memantine can induce cellular proliferation and increase the production of neural progenitors by up to 2–3 times, which may contribute to the regeneration of injured tissues in NMOSD (Cahill et al., 2018).

Another drug that may be helpful in the treatment of NMOSD is dimethyl fumarate (DMF or BG-12). DMF is used to treat relapsing-remitting multiple sclerosis (MS; Höftberger and Lassmann, 2017; Mills et al., 2018). DMF mainly modulates the immunological profiles of patients in terms of cellular composition and inflammation, reducing the number of peripheral T and B lymphocytes and modulating their inflammatory state to engender an anti-inflammatory profile. Further, DMF has a protective effect on cells that suffer from oxidative stress through activation of nuclear factor-erythroid 2-related factor 2 (Nrf2) and can increase the proliferation of neural progenitors *in vitro* since DMF increases self-renewal and protects neural progenitors, oligodendrocytes and therefore myelin against oxidative stress, reducing death by apoptosis (Hammer et al., 2018; Mills et al., 2018). Although some studies show that DMF plays a neuroprotective role, its mechanism of action is still obscure and requires further clarification because many immunomodulatory drugs used in MS treatment may actually worsen NMOSD. Therefore, any trial with DMF should be performed only after strong experimental evidence is found showing that the beneficial effects observed in MS are also observed in NMOSD (Yamout et al., 2017; Popiel et al., 2018).

## Conclusions

The concept that AQP4-IgG auto-antibodies are deleterious to astrocytes is well established. Protein-antibody binding not only activates the immune system but also modulates AQP4 function and induces its internalization by astrocytes. Some pathological features observed in longitudinally extensive myelitis and optic neuritis lesions may be associated with glutamatergic excitotoxicity since the AQP4-IgG/AQP4 complex downregulates the main astrocytic glutamate transporter EAAT2. Consequently, neurons and glial cells may be exposed to excitotoxicity, oxidative stress and neuroinflammation. These three aspects may influence the formation and extension of NMOSD lesions. Drugs to control the deleterious effects of excess glutamate in the CNS may provide innovative neuroprotective therapy to reduce NMOSD attacks and their severity. Therefore, promoting future studies with such drugs in monotherapy or in association with currently used immunotherapies is critical to evaluate the safety and efficacy of these strategies in NMOSD.

## Author Contributions

All authors contributed to the manuscript preparation and wrote, read and approved the submitted version.

## Conflict of Interest Statement

AS has received a scholarship from Conselho Nacional de Desenvolvimento Científico e Tecnológico (CNPq/Brazil). DKS has received a scholarship from the Ministry of Education, Culture, Sports, Science and Technology (MEXT) of Japan; a Grants-in-Aid for Scientific Research from the Japan Society for the Promotion of Science (KAKENHI 15K19472); research support from CNPq/Brasil (425331/2016-4), FAPERGS/MS/CNPq/SESRS (03/2017) PPSUS/Brazil, TEVA (research grant for EMOCEMP Investigator Initiated Study), and Euroimmun AG (Neuroimmunological Complications associated with Arboviruses); and speaker honoraria from Biogen, Novartis, Genzyme, TEVA, Merck-Serono, Roche, and Bayer and has participated in advisory boards for Shire, Roche, TEVA, Merck-Serono and Quest/Athena Diagnostics. The remaining authors declare that the research was conducted in the absence of any commercial or financial relationships that could be construed as a potential conflict of interest.
